# Fabrication of flexible conductive films by rubbing in technology for application in elastic thermo-electric cells

**DOI:** 10.1016/j.mex.2019.02.023

**Published:** 2019-02-26

**Authors:** Khasan S. Karimov, Zubair Ahmad, Jolly Bhadra, N.J. Al-Thani

**Affiliations:** a Ghulam Ishak Khan Institute of Engineering Science & Technology, Topi, District Swabi, Pakistan; b Academy of Sciences of the Republic of Tajikistan, 734015, Rudaki Ave., 33, Dushanbe, Tajikistan; c Center for Advanced Materials (CAM), Qatar University, P.O. Box 2713, Doha, Qatar

**Keywords:** Rubbing technique, Rubbing-in-technology, Flexible conductive thin film, Thermo-electric-cell

## Abstract

This method solves the problem of fabrication of flexible elastic conductive thin film samples for thermoelectric applications. For this purpose, rubbing in technology at room temperature condition has been used which is simple, economical and reliable. As a result, elastic thermo-electric cells have been fabricated that can be used for low power applications and for measurement of the gradient of temperature in industry, medicine and in instrumentation as well. The elastic nature of the thermo-electric cells allows us to place the “hot” and “cold” points of the thermo-electric cells in different planes that make these thermo-electric cells useful for different kind of applications without limitation to place them in a line or in a plane.

## Graphical abstract

**Figure:**
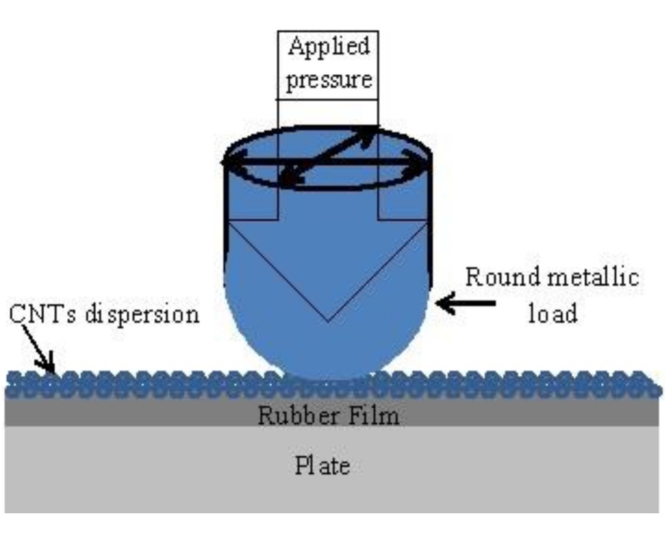

Specifications Table

**Table:** 

**Subject Area:**	• *Energy* • *Engineering* • *Materials Science*
**More specific subject area:**	*Thermo-electric-cell (generator)*
**Method name:**	*rubbing technique*
**Name and reference of original method:**	*rubbing technique* [[1], [2], [3], [4], [5]]
**Resource availability:**	Materials that are used for the fabrication of these thermoelectric cells are economical and are easily available in commercial market. The materials used are: thin rubber films, multiwall carbon nanotubes (CNTs), CNTs composites with some organic and inorganic materials such as copper phthalocyanine (CuPc), nickel phthalocyanine (NiPc), p-type bismuth telluride (p-Bi_2_Te_3_), Poly(3,4-ethylenedioxythiophene)-poly(styrenesulfonate) PEDOT:PSS and silver paste). The selection of the materials has been done based on their potential for flexible thermo-electric cell and generators.

## Method details

**Fig. 1:**
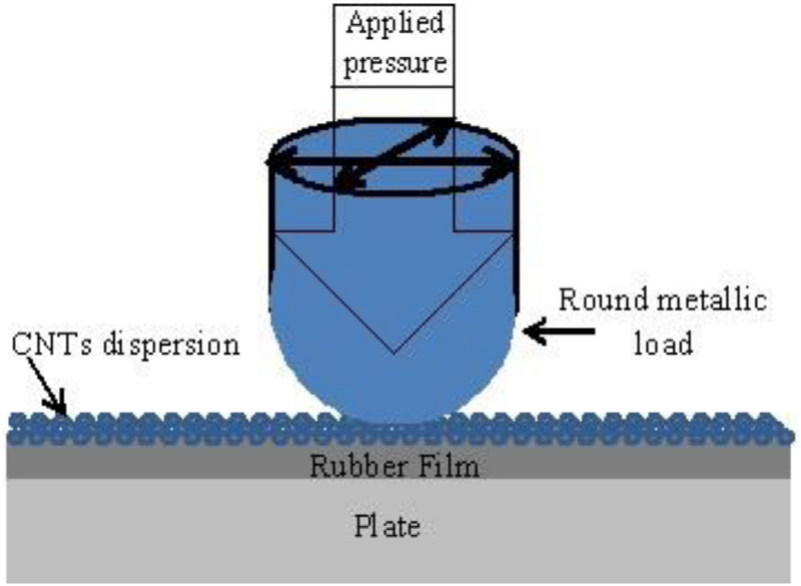
Schematic diagram for the fabrication of flexible elastic conductive films by rubbing in technology for thermo-electric applications.

**Fig. 2:**
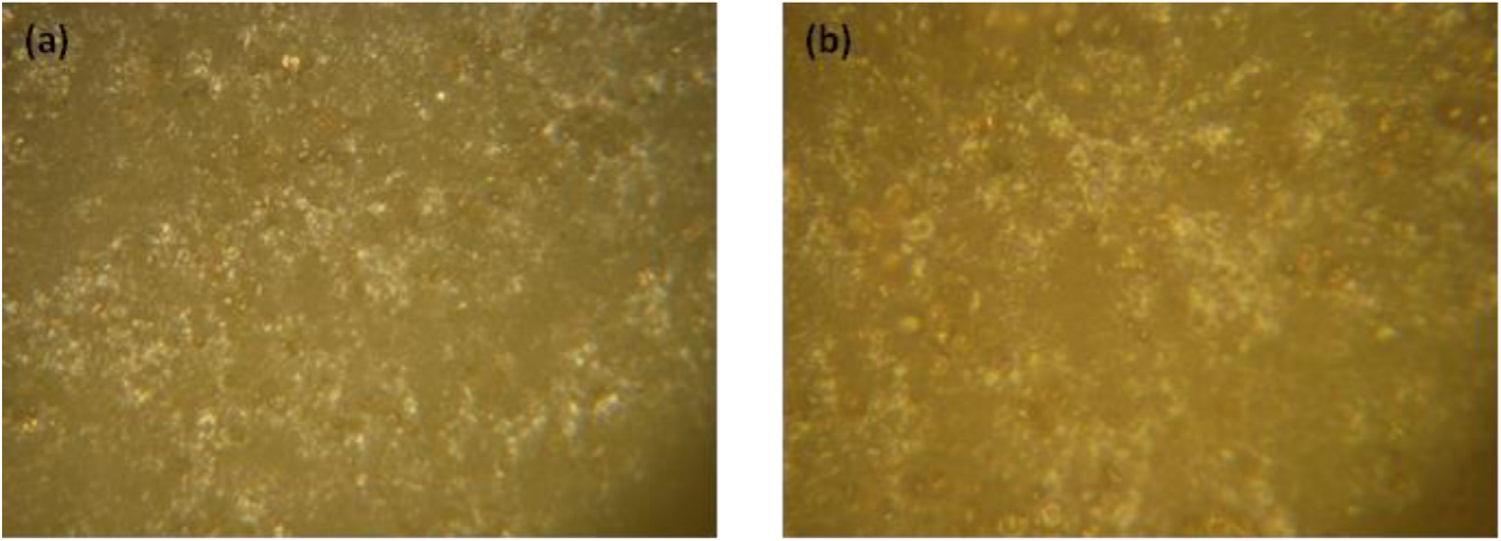
Optical microscope images of the rubber films at strained conditions at magnification of 100 times (a) and 400 times (b).

The new point of the method is the fabrication of the flexible conductive thin films by rubbing in technology that allow to use them in elastic thermo-electric cells. Technology of fabrication is very simple. The technology of thin film fabrication has the following steps: 1) commercially available 2 cm × 2 cm rubber films, the thickness of the film was 80 μm, were fixed on a metallic round plane substrate under the strained condition. Experimentally, it was found that optimal strain (S= Δl*/ l,* where Δl *is* the change in the length (*l*)) is in the range of 1.6–1.8. On the rubber films the powder of nanomaterial or mixed nanomaterials was spread. The polished metallic load was used for rubbing in the nano-powder into the substrate. A special mechanism has been used to drive the load in a horizontal plane at a frequency of (1–10) Hz into two perpendicular directions. Fig. 1 shows the fabrication procedure by rubbing in technology of flexible elastic conductive films for thermo-electric applications. Rubber substrates were fixed on the polished solid plate at strained conditions (strain S = 1.6–1.8). Rubbing in procedure takes (1–3) min. The pressure of the load to the surface of the sample were in the range of (5–10) g/cm^2^. As a result, it was fabricated the samples with active sizes in term of length and width in the range of (5–10) mm and (5–10) mm respectively. It is considered that under the strained condition, the pore sizes in the rubber substrates increased. This makes it easier to realize the rubbing-in technology to embed the nano-powder into the rubber substrate to develop the conductive flexible elastic substrates at room temperature. Fig. 2(a) and (b) show the optical microscopic images of the rubber films at strained conditions (strain S = 1.6) at magnifications of 100 times (a) and 400 times (b). After following the above-mentioned procedure, the stress has been released to let the pore sizes to decrease which enables the nanoparticles to squeeze. These embedded films showed very stable electrical properties even at the temperature range from (30–70) ^o^C which is very important in the case of thermo-electric applications.

**Fig. 3:**
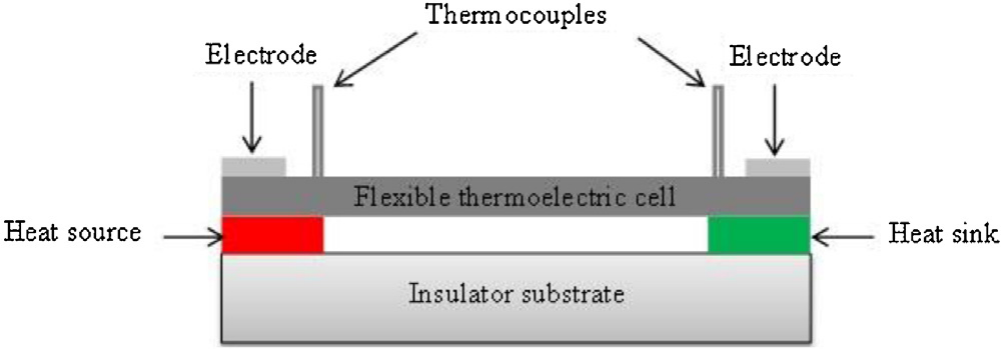
Schematic diagram of the thermo-electric properties measurement setup for the flexible thermo-electric cell.

Fig. 3 shows the schematic diagram of the thermo-electric properties measurement setup for the flexible termo-electric cell. By using this technology seven different samples using CNTs, CNTs-NiPc, CNTs-CuPc, CNTs-p-Bi_2_Te_3_, CNTs-silver paste and CNT-PEDOT:PSS composites were fabricated and characterized. Seebeck coefficient (α) of the samples was calculated by using the following Eq. (1):

(1) $$\alpha =V_{oc}/\Delta T$$

where $V_{oc}$ and ΔT are the generated thermo-electric open-circuit voltages and gradient of temperature, respectively. In this experiment the gradient of temperature was kept at 5 °C.

Furthermore, CNTs were also deposited on the both sides of the rubber film by rubbing in technology and it was found that the short circuit current increased approximately two times and the internal resistance of the thermo-electric cells decreased accordingly, whereas, thermo-electric open-circuit voltage remained the same. Table 1 presents the thermo-electric properties of flexible thermo-electric cells.

**Table 1:** The obtained results of flexible thermo-electric cells fabricated by rubbing in technology.

No.	Composition of the deposited nano structured materials on the elastic rubber substrate	V_oc_ (μV)	α (μV/^o^C)	I_sc_ (μA)	*I_n_(μA)	R(kΩ)
1	CNTs one side of the substrate**	175	35	0.017	0.01	15
2	CNTs on both sides of the substrate**	175	35	0.035	0.016	8
3	CNTs:NiPc) (3:2 by weight) **	225	45	0.003	0.001	86
4	CNTs: CuPc (3:2 by weight) **	190	38	0.002	0.001	99
5	CNTs:p-Bi_2_Te_3_ (1:1 by weight) ***	255	51	0.003	0.002	94
6	CNTs: silver paste (1:1 by weight) ***	160	32	0.046	0.023	3.5
7	CNTs: PEDOT:PSS (1:1 by weight) ***	180	36	0.004	0.002	41

I_sc_ (μA)- short-circuit current, *I_n_(μA)- is the current at resistive load that is equal to the internal resistance of the cell, R(kΩ) – resistance of the samples.

The proposed rubbing in method is realized at room temperature condition and normal atmospheric pressure for fabrication of flexible elastic conductive films, unlike the deposition of metallic films on rubber films at vacuum deposition or at deposition at elevated temperatures. This method can be used for fabrication of flexible elastic conductive films. Moreover, flexible elastic thermo-electric cells can be fabricated using these conductive films to be used for low power application and measurement of the gradient of temperature in industry, medicine, and instrumentation. The limitation of this method is the selection of the proper rubber substrate. However, this limitation can be easy to overcome. If the substrate consists of dense rubber, then nanoparticles should be used. If the sizes of particles are in the micrometer range, then the rubber substrate should have accordingly larger sizes of pores.
